# Evaluating Antibody Mediated Protection against Alpha, Beta, and Delta SARS-CoV-2 Variants of Concern in K18-hACE2 Transgenic Mice

**DOI:** 10.1128/jvi.02184-21

**Published:** 2022-03-23

**Authors:** Ting Y. Wong, Alexander M. Horspool, Brynnan P. Russ, Chengjin Ye, Katherine S. Lee, Michael T. Winters, Justin R. Bevere, Olivia A. Miller, Nathaniel A. Rader, Melissa Cooper, Theodore Kieffer, Julien Sourimant, Alexander L. Greninger, Richard K. Plemper, James Denvir, Holly A. Cyphert, Mariette Barbier, Jordi B. Torrelles, Ivan Martinez, Luis Martinez-Sobrido, F. Heath Damron

**Affiliations:** a Department of Microbiology, Immunology, and Cell Biology, West Virginia University, Morgantown, West Virginia, USA; b Vaccine Development Center at West, Virginia University Health Sciences Center, Morgantown, West Virginia, USA; c West Virginia University Cancer Institute, Morgantown, West Virginia, USA; d Department of Pathology, Anatomy and Laboratory Medicine, West Virginia University School of Medicine, Morgantown, West Virginia, USA; e Institute for Biomedical Sciences, Georgia State University, Atlanta, Georgia, USA; f Department of Laboratory Medicine and Pathology, University of Washington, Seattle, Washington, USA; g Department of Biomedical Sciences, Marshall University, Huntington, West Virginia, USA; h Department of Biological Sciences, Marshall University, Huntington, West Virginia, USA; i Host-Pathogen Interactions and Population Health Programs, Texas Biomedical Research Institute, San Antonio, Texas, USA; Loyola University Chicago

**Keywords:** SARS-CoV-2, COVID-19, variants of concern, Alpha, Beta, Delta, K18-hACE2 transgenic mice, convalescent plasma, modeling COVID-19, passive immunity

## Abstract

SARS-CoV-2 variants of concern (VoC) are impacting responses to the COVID-19 pandemic. Here, we utilized passive immunization using human convalescent plasma (HCP) obtained from a critically ill COVID-19 patient in the early pandemic to study the efficacy of polyclonal antibodies generated to ancestral SARS-CoV-2 against the Alpha, Beta, and Delta VoC in the K18 human angiotensin converting enzyme 2 (hACE2) transgenic mouse model. HCP protected mice from challenge with the original WA-1 SARS-CoV-2 strain; however, only partially protected mice challenged with the Alpha VoC (60% survival) and failed to save Beta challenged mice from succumbing to disease. HCP treatment groups had elevated receptor binding domain (RBD) and nucleocapsid IgG titers in the serum; however, Beta VoC viral RNA burden in the lung and brain was not decreased due to HCP treatment. While mice could be protected from WA-1 or Alpha challenge with a single dose of HCP, six doses of HCP could not decrease mortality of Delta challenged mice. Overall, these data demonstrate that VoC have enhanced immune evasion and this work underscores the need for *in vivo* models to evaluate future emerging strains.

**IMPORTANCE** Emerging SARS-CoV-2 VoC are posing new problems regarding vaccine and monoclonal antibody efficacy. To better understand immune evasion tactics of the VoC, we utilized passive immunization to study the effect of early-pandemic SARS-CoV-2 HCP against, Alpha, Beta, and Delta VoC. We observed that HCP from a human infected with the original SARS-CoV-2 was unable to control lethality of Alpha, Beta, or Delta VoC in the K18-hACE2 transgenic mouse model of SARS-CoV-2 infection. Our findings demonstrate that passive immunization can be used as a model to evaluate immune evasion of emerging VoC strains.

## INTRODUCTION

The evolution of Severe Acute Respiratory Syndrome CoV-2 (SARS-CoV-2) variants of concern (VoC) has been a source of escalating epidemiological alarm in the currently ongoing coronavirus disease 2019 (COVID-19) pandemic. SARS-CoV-2 VoC have emerged and are thought to be more infectious and more lethal than the early 2020 original Wuhan-Hu-1 or USA-WA1/2020 (WA-1) strains (1–3). The VoC B.1.1.7, also known as Alpha variant (first identified in the United Kingdom) (4), and B.1.351 also known as Beta variant (first identified in South Africa) (5), were two SARS-CoV-2 VoC that rapidly spread around the world and exhibited high levels of infectivity and therapeutic resistance (3, 6–11). Both VoC contain important mutations in the receptor binding domain (RBD) of the spike (S) viral glycoprotein (4, 5) that are predicted to impact binding to the human angiotensin converting enzyme 2 (hACE2) viral receptor and enhance viral entry into host cells (12–16). In particular, Alpha contains the D614G and N501Y mutations in the SARS-CoV-2 S RBD which are theorized to increase the ability of the virus to bind to hACE2 (12, 14). Beta possesses these key mutations in the S RBD, in addition to the K417N and E484K mutations which are not directly implicated in altered viral transmission and hACE2 binding (16, 17). In December 2020, the VoC, B.1.617.2 (Delta) of SARS-CoV-2 first appeared in India, becoming quickly the global predominant circulating variant; however, this distinction could be soon displaced by the novel Omicron variant (18–20). The most common Delta variant has two important mutations on the viral S RBD, L452R and T478K, allowing for increased infectivity, transmissibility, as well as its ability of escaping neutralizing antibodies (21–23). The culmination of high infectivity, therapeutic resistance, and key changes in their viral genome suggests that VoC may have an impact on pathogenicity in animal models of SARS-CoV-2, with a subsequent impact on evaluating vaccines and therapeutics.

The K18-hACE2 transgenic mouse model (24) of SARS-CoV-2 infection was established by several groups in 2020 (25–27). K18-hACE2 transgenic mice challenged with SARS-CoV-2 exhibited significant morbidity and mortality, viral tropism of the respiratory and central nervous systems, elevated systemic chemokine and cytokine levels, significant tissue pathologies, and altered gross clinical measures (26–29). The generation of this mouse model has led to numerous studies of SARS-CoV-2 challenge for a variety of purposes including understanding SARS-CoV-2 related immunity, and therapeutic/vaccine testing (25, 30–35). As the world experiences an increase in the number of SARS-CoV-2 VoC, it is imperative to adapt existing preclinical animal infection models to these newly emerging VoC. Specifically, it is critical to understand if the K18-hACE2 transgenic mouse model first, is useful for studying SARS-CoV-2 VoC infection dynamics and second, if it exhibits any differences after challenge with newly emerged SARS-CoV-2 VoC. An investigation of these key points will provide context for studies important for developing new therapeutics and prophylactics as the COVID-19 pandemic continues and as new VoC emerge.

Neutralizing antibodies against SARS-CoV-2 induced by either natural infection or vaccination serve as an important component of protection against secondary SARS-CoV-2 infection (36); however, according to the WHO and recent data, Omicron variant appears to be able to easily infect fully vaccinated individuals. The S protein is a major target of neutralizing antibodies, with RBD encompassing 90% of the neutralizing antibodies within convalescent-phase sera (37, 38). Emergence of new VoC with mutations in the S protein and in the RBD could decrease the efficacy of neutralizing antibodies not originally generated against the VoC. Studies have shown that *N*-terminal domain S and RBD monoclonal antibodies generated against the original SARS-CoV-2 strain lose neutralization activity against VoC especially when administered as a monotherapy (7, 9, 39). Human convalescent plasma (HCP) also has demonstrated a decrease in neutralizing antibody efficacy against the VoC that specifically harbor the E484K mutation in the S RBD (7, 9). Here, we evaluated the polyclonal antibodies of HCP obtained from a patient infected with the original strain of SARS-CoV-2 against the Alpha, Beta, and Delta VoC in the K18-hACE2 transgenic mouse model. Our findings indicate that when compared with the original WA-1 strain, Alpha, Beta and Delta VoC are more resistant to HCP polyclonal antibodies in the K18-hACE2 transgenic mouse model. This passive immunity model allows for comparison of *in vivo* activity of human antibodies, extends upon *in vitro* studies and will likely assist in understanding immunity among VoC.

## RESULTS

### Evaluating human antibodies against original SARS-CoV-2 for their ability to protect VoC challenged mice.

**FIG 1:**
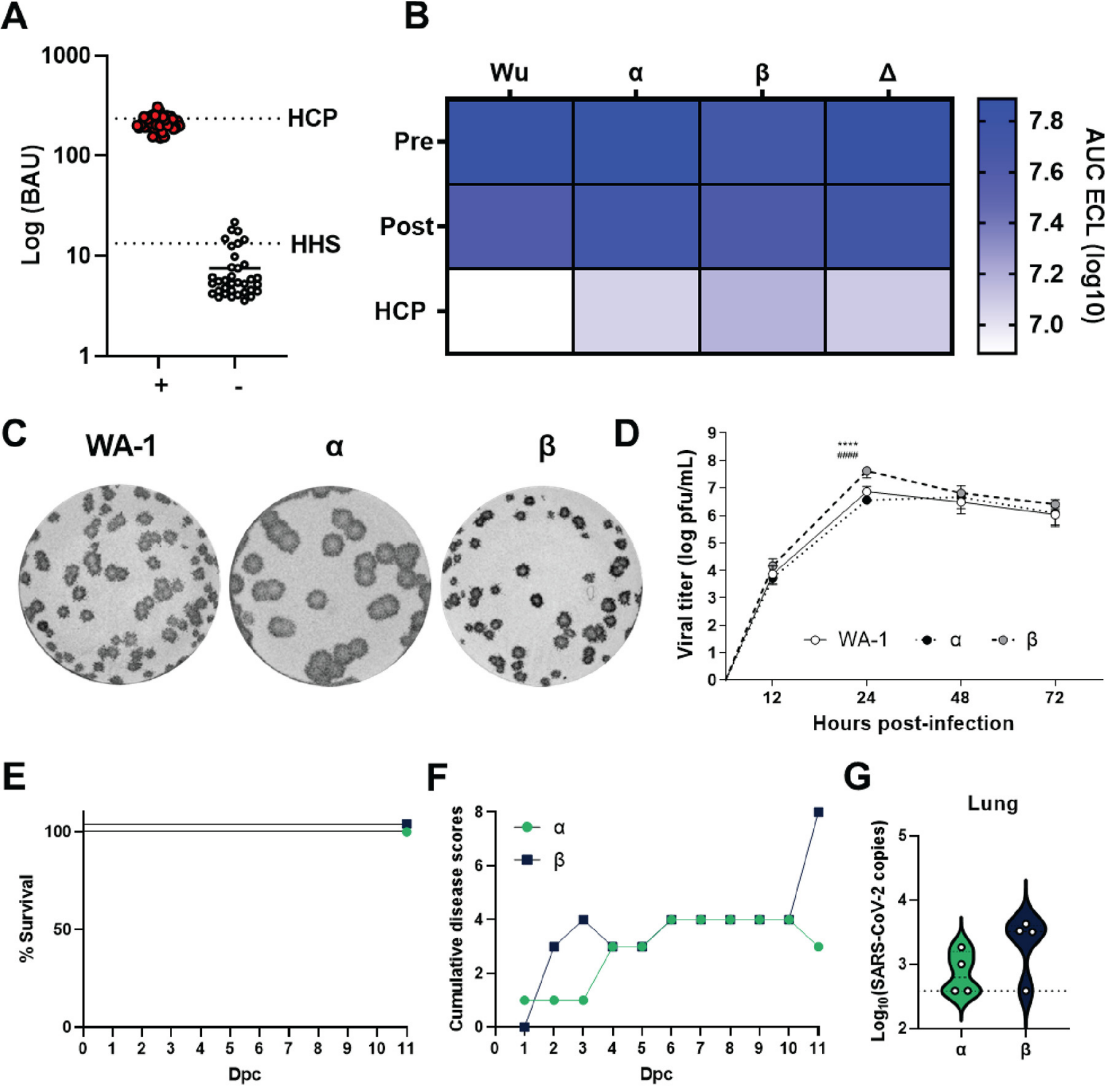
Characterization of early pandemic human convalescent plasma and *in vitro* characterization of SARS-CoV-2 variants. (A) RBD human IgG Binding antibody units (BAU) of SARS-CoV-2 + (red dots) compared to SARS-CoV-2 – patients (white dots). HCP dotted line indicate the BAU of the human convalescent plasma from a severe COVID-19 patient utilized in passive immunization studies in K18-hACE2 transgenic mice. HHS dotted line indicate the BAU of the healthy human serum used in passive immunization studies in K18-hACE2 transgenic mice. (B) ACE2-RBD neutralization was assayed, and the human convalescent plasma utilized was more capable of neutralizing receptor binding than mRNA vaccinated human sera. The heat map depicts the log_10_ AUC of electro chemiluminescent (ECL) values. (C) Plaque morphology of SARS-CoV-2 WA-1, Alpha or Beta infected VeroE6 cells. (D) Viral replication of SARS-CoV-2 variants in VeroE6 cells over time was quantified. Statistical analysis of viral replication was completed by two-way ANOVA followed by Tukey’s multiple comparison test, or RM ANOVA followed by Tukey’s multiple comparison test. **** = *P < *0.0001 relative to WA-1, ^####^ = *P < *0.0001 relative to Alpha. C57BL6/J Mice were infected with 10^5^ PFU SARS-CoV-2 VoC monitored for survival (E) and disease score (F). (G) Challenge with Alpha or Beta variants resulted in low detectable virus at day 11 post challenge. Dotted line represents limit of detection.

The emergence of SARS-CoV-2 VoC requires re-investigation of their pathogenesis and unique properties. Our goal for this part of the study was to determine if ancestral virus specific antibodies raised in humans would be able to provide protection against Alpha and Beta VoC challenge in K18-hACE2-mouse challenge model. HCP was extensively used early in the COVID-19 pandemic, but currently it is no longer used as a standard of care. The selected HCP for these studies originated from a patient with severe COVID-19 disease in 2020 and contained 236 antibody binding units (WHO COVID-19 International Standard; BAU). This HCP was compared with other 48 HCP samples from COVID-19 patients taken back in spring of 2020 (Fig. 1A). Next, the selected HCP was compared with serum obtained from pre-vaccine and post Pfizer mRNA vaccinated healthy volunteers. The selected HCP sample was able to neutralize Wuhan, Alpha, Beta, and Delta RBD to ACE2 binding using the MSD hACE2-RBD *in vitro* neutralization assay (Fig. 1B). These data indicate that the selected HCP had high binding and neutralization capacity. *In vitro* cell culture growth experiments were performed to characterize the Alpha and Beta VoC. The Beta variant appeared to have a modest increase in PFU/ml after 24 h of growth *in vitro* (Fig. 1CD); however, it had a relatively similar growth curve compared to the original WA-1 strain and Alpha VoC. One caveat about using Alpha or Beta challenge strains in mice, is that it is possible the mutations in RBD will allow for binding and engagement of the mouse ACE2 receptor. Mouse adapted SARS-CoV-2 strains are used to challenge wild type, non-transgenic mice (40), and VoC strains are known to replicate in wild-type mice (41). We performed a challenge study with Alpha and Beta VoC in wild type C57BL6/J mice; however, morbidity or mortality was not observed (Fig. 1E). We observed low disease scores, and very little detectable viral RNA in the lungs of the wild type challenged mice (Fig. 1FG). Based on these data, we do not believe there is much concern about using Alpha or Beta in mice because it appears their ability to infect through mouse ACE2 is limited.

### Effects of HCP treatment on disease progression in mice challenged with SARS-CoV-2 VoC.

**FIG 2:**
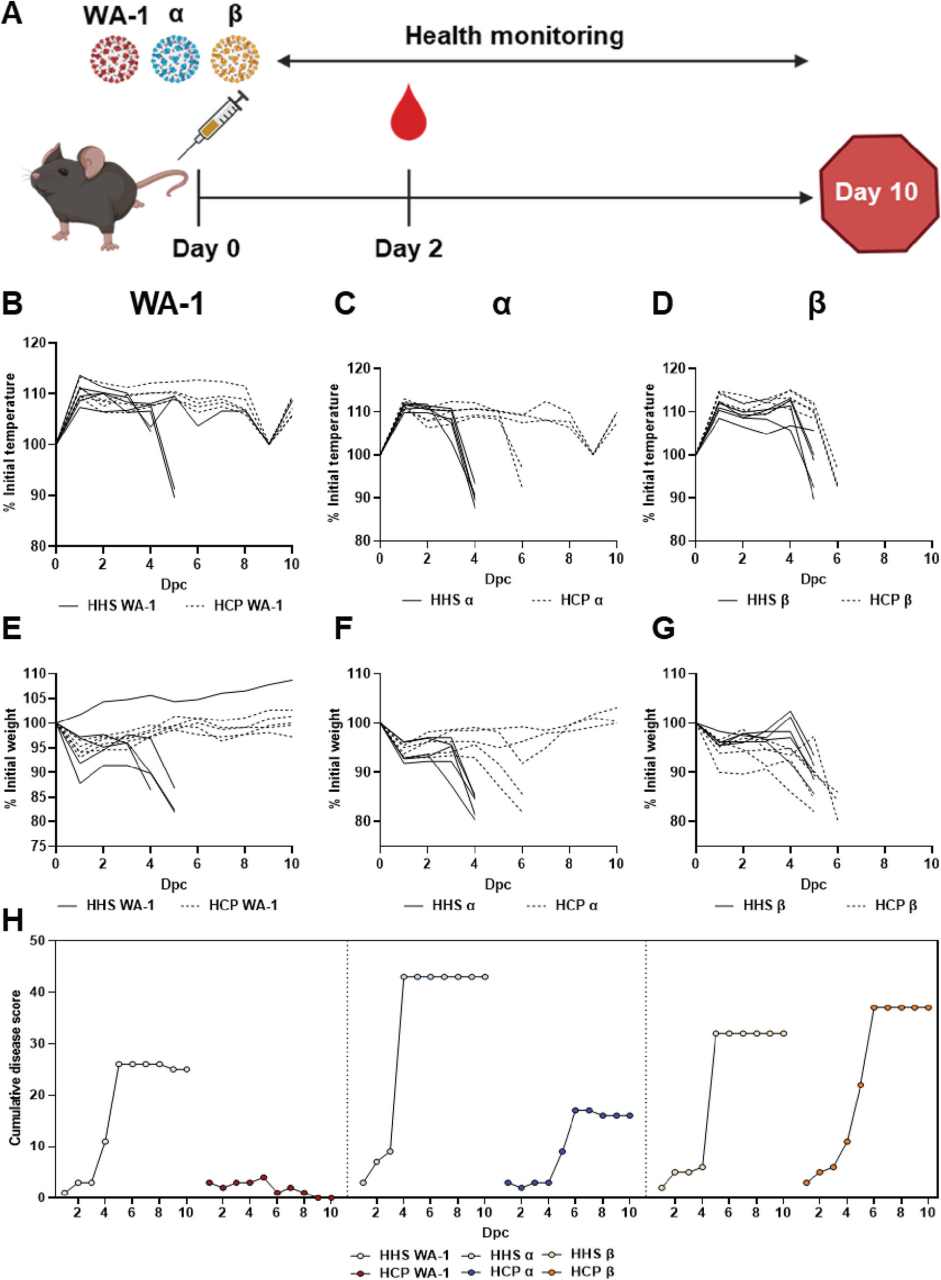
Effect of convalescent plasma treatment on SARS-CoV-2 VoC challenge in K18-hACE2 transgenic mice. (A) Passive immunization and SARS-CoV-2 challenge schematic. Mice were challenged with 10^5^ PFU of SARS-CoV-2 WA-1 and VoC and simultaneously treated intraperitoneally with 500 μl HHS or HCP on day 0. Mice were monitored for temperature (B-D), body weight (E-G) and cumulative clinical score (H) over the 7-day course of infection.

**FIG 3:**
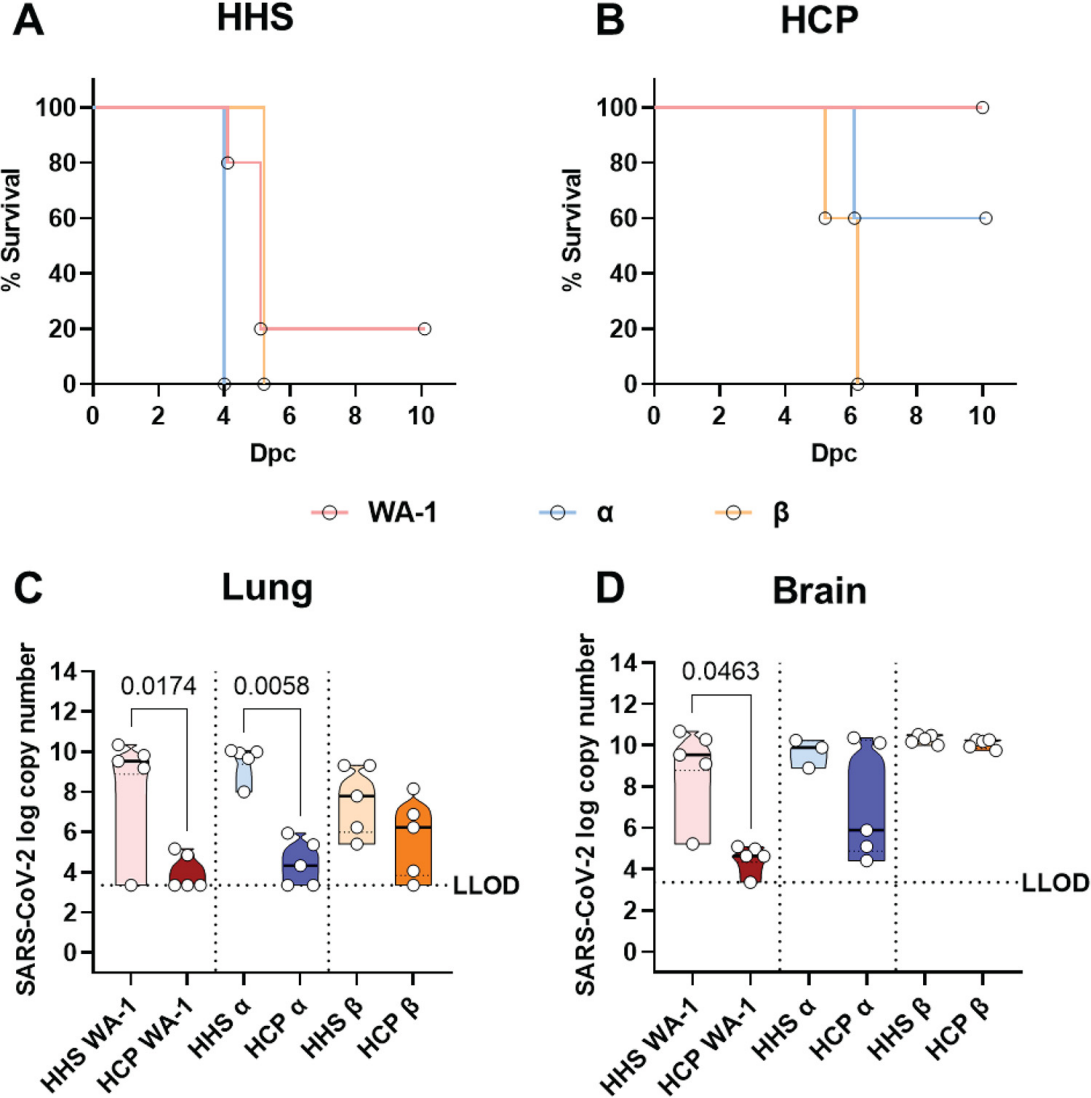
Survival and viral RNA burden of serum-treated K18-hACE2 transgenic mice challenged with SARS-CoV-2 VoC. Kaplan-Meyer survival curves of mice challenged with Alpha, Beta, or WA-1 treated with HHS (A) or early pandemic SARS-CoV-2 HCP (B). Viral copy numbers in the lung (C) and brain (D) of challenged mice. LLOD = lower limit of detection based on a standard curve. Statistical significance of survival curves was assessed with the Mantel-Cox test. For HHS, WA-1 vs Alpha *P = *0.0143; WA-1 vs Beta *P* = 0.9372 and Alpha vs Beta *P* = 0.0027. For HCP, WA-1 vs Alpha *P = *0.1336; WA-1 vs Beta *P* = 0.0031 and Alpha vs Beta *P* = 0.0290. Statistical significance between viral copy number was assessed by a Kruskal-Wallis test followed by Dunn’s multiple-comparison test. *n* > 3 subjects per group. *P values* for significant differences are reported.

K18-hACE2 transgenic mice were passively immunized with HCP via intraperitoneal administration at day 0 and subsequently challenged with 10^5^ PFU (lethal dose) of WA-1, Alpha, or Beta VoC (Fig. 2A). WA-1 challenged mice that received human serum from healthy individuals (HHS) exhibited a temperature drop, weight loss, and high cumulative disease scores (Fig. 2B, E, H). Mice treated with HCP had normal temperature regulation, maintained weight, and had low disease scores (Fig. 2B, E, H). Protection from WA-1 lethal challenge in HCP treated mice was expected since convalescent humans have immunity against re-challenge. Challenge with Alpha VoC in HHS treated mice resulted in high temperature loss by day 4 post challenge, up to 20% weight loss, and high cumulative disease scores (Fig. 2C, F, H). However, Alpha VoC challenged mice treated with HCP maintained body temperature in three of five animals and similar trends were observed for their body weight loss (Fig. 2CF). These data suggested that HCP was less successful at protecting mice from Alpha VoC challenge compared to WA-1. Disease scores also reflected these observations as HCP treatment was unable to fully suppress disease (Fig. 2H). Unlike WA-1 or Alpha VoC challenged mice, Beta VoC challenged mice treated with HCP compared to HHS had no significant differences by any metric measured (Fig. 2D, G, H). HCP treatment was unsuccessful in preventing disease and morbidity induced by the Beta VoC. Collectively, these data showed that HCP treatment was able to fully protect against WA-1; partially protect against Alpha VoC; but failed to protect against Beta VoC (Fig. 3AB).

### Effects of HCP treatment on viral RNA burden in lungs and brain of challenged mice.

To determine the viral distribution between the lungs and brain of challenged mice, qRT-PCR was used to quantify nucleocapsid copy number. HCP treatment significantly decreased viral RNA down to the lower limit of detection in the lung of the WA-1 and Alpha challenged treated with HHS mice compared to HHS (Fig. 3C). Beta variant challenged mice had two logs lower RNA compared to WA-1 and Alpha HHS treated mice and HCP treatment was able to decrease two of the mice down to the lower limit of detection (Fig. 3C). A lethal dose of SARS-CoV-2 WA-1 is known to infiltrate the brain of K18-hACE2 transgenic mice (26, 27). As expected, brain WA-1 viral copy numbers were decreased due to HCP treatment (Fig. 3D). Similarly, three of five Alpha VoC challenged mice treated with HCP had low viral RNA detected in their brains (Fig. 3D), which corresponded to their survival data (Fig. 3AB). Surprisingly, HCP treatment did not decrease brain Beta VoC virus RNA copies, further demonstrating the ability of Beta VoC to break through antibody protection that was derived against original Wuhan or WA-1-like viruses (Fig. 3D).

### Human IgG levels in convalescent plasma treated K18-hACE2 transgenic mice challenged with SARS-CoV-2 VoC.

**FIG 4:**
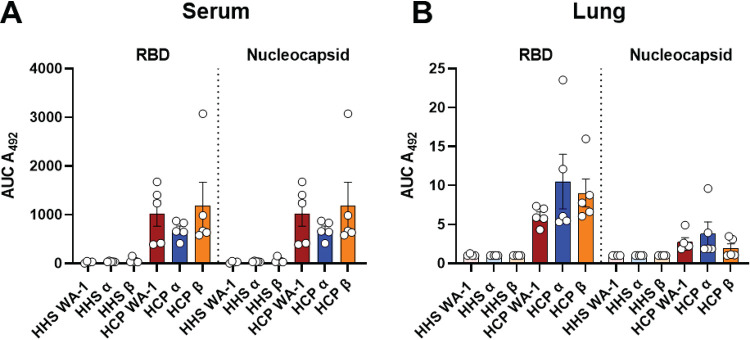
Human anti-SARS-CoV-2 IgGs in serum-treated K18-hACE2 transgenic mice challenged with SARS-CoV-2 VoC at euthanasia. Area under the curve (AUC) analyses of anti-RBD IgG levels in the serum (A) or lung (B) of HHS or HCP VoC challenged mice. Statistical significance between AUCs was assessed by a Kruskal-Wallis test followed by Dunn’s multiple-comparison test. *n* > 3 subjects per group.

To determine the level of IgGs delivered to HHS and HCP treated mice, we analyzed whether human anti-SARS-CoV-2 IgGs were present within the lung and sera of animals treated with HCP or HHS through the course of infection (Fig. 4AB). Data demonstrated that significant quantities of human anti-SARS-CoV-2 IgGs targeting both the RBD and nucleocapsid proteins were present at 2 days post challenge in HCP-treated relative to HHS-treated mice (data not shown) as well as at euthanasia in the sera and lung (Fig. 4AB). Overall, these data indicated that passive immunization with HCP resulted in persistence of SARS-CoV-2 specific human antibodies in mice through the experimental time frame studied.

### HCP treatment lowered chronic and acute inflammation in the lung caused by SARS-CoV-2 challenge.

**FIG 5:**
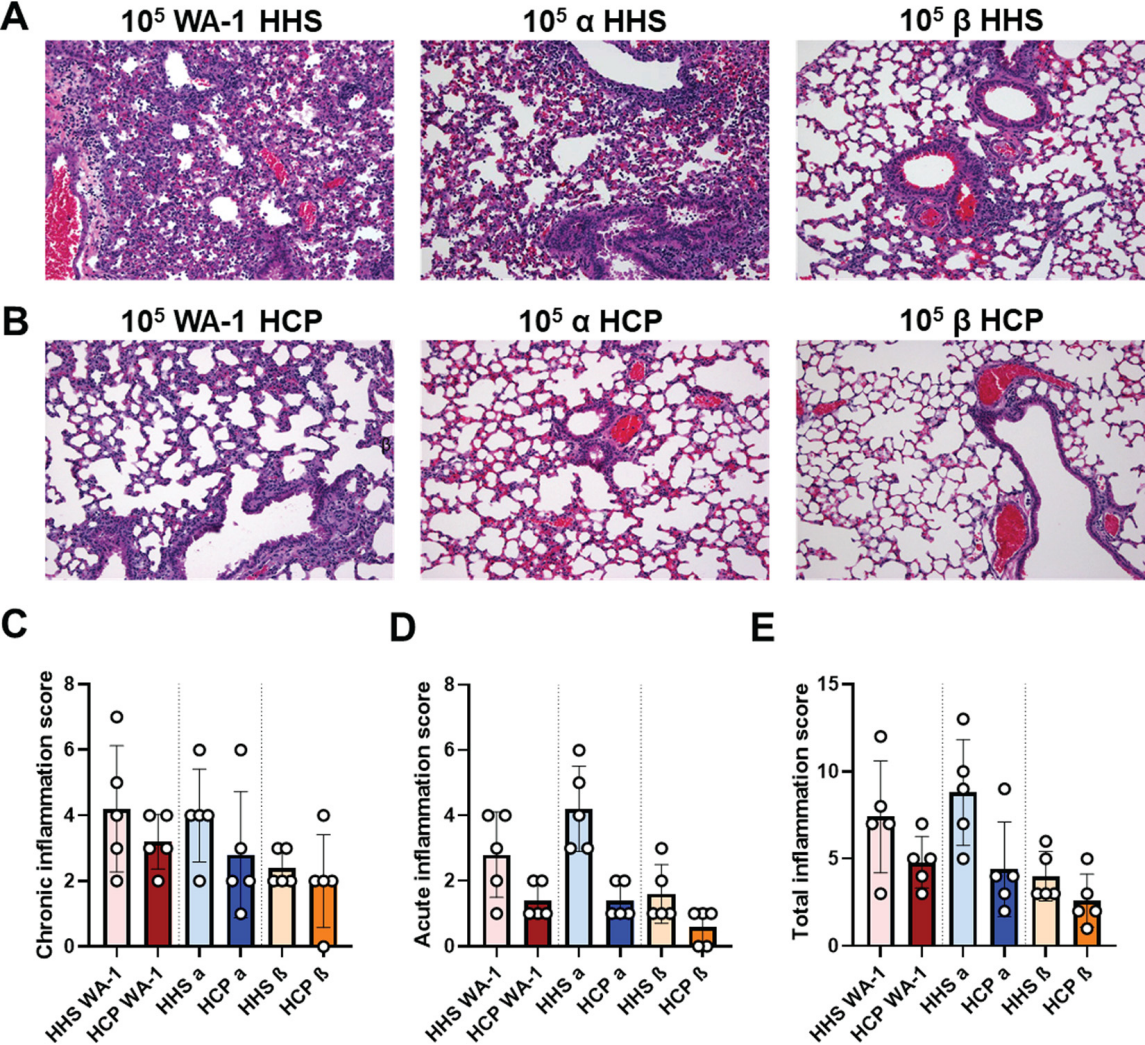
Histopathological analysis of VoC challenged lungs. Left lobes of lungs from HHS and HCP treated and SARS-CoV-2 challenged mice were subjected to hematoxylin and eosin staining (A) ×200 magnification of the lung in HHS treated and SARS-CoV-2 challenged mice (B) ×200 magnification of the lung in HCP treated and SARS-CoV-2 challenged mice (C) Total chronic inflammation scores of each mouse. (D) Total acute inflammation score of each mouse. (E) Total inflammation score (chronic + acute) for each mouse. All statistical analysis was performed using Kruskal-Wallis test with Dunn’s multiple-comparison test.

Histopathology analysis was performed to characterize disease manifestation in the lung due to inflammation caused by WA-1, Alpha, or Beta challenge during HHS and HCP treatments (Fig. 5). Chronic inflammation was denoted as presence of lymphocytes, plasma cells and alveolar macrophages, whereas acute inflammation was characterized by neutrophils and edema in the lung parenchyma, vasculature, and bronchi. Total inflammation was determined by the addition of chronic and acute inflammation scores. HHS treatment groups challenged with WA-1, Alpha or Beta VoC had the highest chronic and acute inflammation scores in the lung parenchyma and surrounding blood vessels compared to the HCP treated mice (Fig. 5A, C–E). HHS treated mice challenged with WA-1 or Alpha VoC had the highest average total inflammation scores of 7.4 and 8.8, respectively; whereas Beta VoC challenged mice had an average total inflammation score of 4.0 (Fig. 5C, D, E). HCP treatment groups challenged with WA-1, Alpha or Beta VoC also had mixed chronic and acute inflammation albeit lower total inflammation compared to HHS treated mice (Fig. 5B–E). HCP treated mice challenged with WA-1 had the highest average total inflammation score (4.0), characterized by more chronic inflammation than acute (Fig. 5C, D). Mice treated with HCP and challenged with Alpha VoC had an average inflammation score of 4.4 and decreased acute inflammation compared to HHS treatment (Fig. 5D-E). Interestingly, HHS and HCP treated mice challenged with Beta VoC had low lung inflammation (Fig. 5C-E), which correlated with the low viral RNA burden of Beta VoC (Fig. 3C). Overall, HHS treated, and SARS-CoV-2 challenged mice had elevated levels of both chronic and acute inflammation compared to the HCP treated and challenged mice.

### HCP passive immunization was insufficient to protect against Delta VoC challenge.

**FIG 6:**
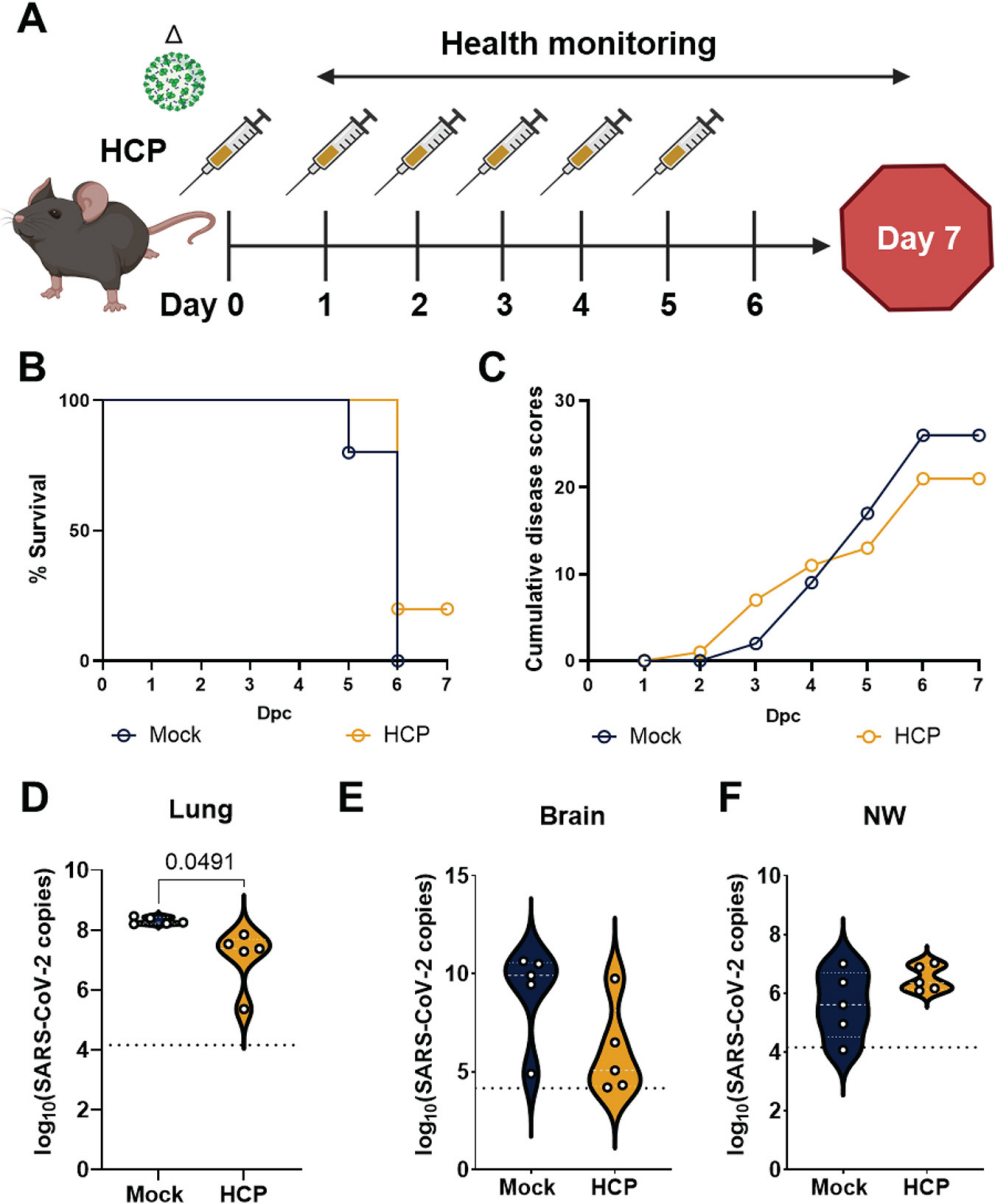
HCP passive immunization was insufficient to protect against Delta variant challenge. (A) Experimental workflow of passive immunization study with HCP and challenge with lethal dose of Delta variant (10^4^ PFU/dose). (B) Kaplan Meier survival curve comparing Delta challenged mice that received either 1XDPBS vehicle or HCP. (C) Cumulative disease scores comparing Delta challenged mice that received either 1XDPBS vehicle or HCP. SARS-CoV-2 nucleocapsid RNA copies in A) lung, B) brain, and C) nasal wash of untreated and HCP treated and challenged mice. All statistical analysis was performed using Welch’s *t* test. *P = *0.0491 (lumg, D), P = 0.0692 (brain, E), and P = 0.1603 (NW, F).

Delta VoC contains mutations on the RBD that compromise antibody neutralization (23). We further evaluated whether polyclonal antibodies in the HCP generated from an original virus immune plasma could protect mice from a lethal Delta VoC challenge. Here, we used a challenge dose of 10^4^ PFU/dose of Delta VoC instead of a 10^5^ PFU/dose as we previously used for WA-1, Alpha, and Beta VoC. In pilot studies, we demonstrated that 10^4^ PFU/dose of Delta VoC resulted in 100% morbidity in K18-hACE2 transgenic mice (data not shown). Thus, mice were administered HCP (*n* = 5) or PBS (*n* = 5) intraperitoneally and concurrently intranasally challenged with a lethal Delta VoC dose on day 0 (Fig. 6A). HCP treated mice received treatment for 5 consecutive days after the first dose on day 0. All mice were monitored for disease for 7 days (Fig. 6A). Mice that did not receive HCP treatment succumbed to Delta VoC challenge by day 6 and had elevated cumulative disease scores (Fig. 6B, C). However, only 20% of mice that received 6 treatments of HCP survived the Delta VoC challenge and had disease scores similar to untreated mice (Fig. 6B, C). Viral RNA burden mirrored survival and disease scores for both HCP treated and untreated mice. Lung, brain, and nasal wash (NW) of the HCP treated mice had similar levels of viral RNA compared to untreated mice indicating that HCP treatment did not block viral replication (Fig. 6D–F). Overall, polyclonal antibodies generated against the ancestral SARS-CoV-2 strain did not protect mice from Delta VoC challenge suggesting that the Delta VoC is resistant to polyclonal antibodies generated against Wuhan-lineage virus strains.

## DISCUSSION

SARS-CoV-2 VoC are constantly evolving and dramatically impacting the ongoing COVID-19 pandemic. Since the beginning of the pandemic, three major infection waves have occurred: (i) original virus, (ii) Alpha variant, and (iii) Delta variant, with a recent wave starting directed by the novel Omicron (B.1.1.529) VoC. Approved vaccines are implemented all around the world with 8 billion total doses administered meaning 1 dose per person in the world. However, there are massive inequities in vaccine coverage with the United States/Canada, Latin America, Asia-Pacific, and Europe with ∼60–70% vaccination with one dose, whereas Africa is only at 10% coverage with one dose. Overall, the world is at 56% vaccine coverage with one dose. All current vaccines are designed against the original virus spike antigen sequence, but two major waves have been fueled by the Alpha and Delta VoC. Vaccine redevelopment will always be a challenge and new VoC have been constantly arising.

Alpha and Beta VoC spike antigens were extensively studied by binding and neutralization assays that suggested antibodies generated by infection or vaccination would be able to provide protection. Ultimately relatively low numbers of vaccine breakthrough occurred. To confirm the *in vitro* predictions regarding Alpha and Beta VoC, we designed this study to use a passive immunization model in K18-hACE2 transgenic mice to compare antibody dependent immunity between original virus *versus* VoC. Our observations suggested that Alpha VoC is partially neutralized in K18-hACE2 transgenic mice treated with HCP (Fig. 2 and 3), where the Beta VoC was not sufficiently neutralized to prevent lethality in this model system (Fig. 2 and 3). HCP treatment dramatically decreased viral RNA burden in the lungs and brain in WA-1 and Alpha VoC challenged mice, but minimal to no decrease was observed in mice challenged by the Beta VoC (Fig. 3C and D), which likely contributed to the morbidity and mortality caused by the Beta VoC. Low viral burden in the lung correlated with low chronic or acute inflammations scores (Fig. 5). Antibody breakthrough and aggressive pathogenesis suggested that the Beta VoC was going to likely be a variant of high concern. When Beta variant appeared, it was able to impact vaccine trial efficacy studies and seemed poised to infect vaccinated people (42–44). However, the Beta VoC peaked at a total of 12% genome worldwide frequency by April 2021. Thus, it seems likely that the Beta VoC was not highly transmissible, and our passive immunization model does not take this variable into consideration.

HCP as a treatment was used widely since the onset of the COVID-19 pandemic (45–47), but its efficacy was questionable (48–51) and convalescent plasma therapy for COVID-19 has been largely replaced as a treatment by monoclonal antibodies. In this study, we used HCP from an early pandemic COVID-19+ severe disease patient to understand how antibodies generated against the original SARS-CoV-2 strain would function against Alpha and Beta VoC. In December 2020, the Delta VoC appeared in India and by mid-2021, this VoC was the dominant variant found in genomic surveillance. To build upon our observations regarding Alpha and Beta VoC in the HCP passive immunity model with K18-hACE2 transgenic mice, we next aimed to evaluate the Delta VoC. Our pilot studies indicated a massive histopathological and inflammatory gene expression in Delta VoC *versus* Alpha VoC challenged mice (data not shown). We reasoned that the Delta VoC was more aggressive, would likely need a lower dose to be fully virulent compared to WA-1 strain and would also require more HCP to neutralize the virus *in vivo*. Thus, we challenged mice with a lower dose of 10^4^ PFU and HCP treatment was provided daily out to 6-days post challenge. Unexpectedly, even though we provided 6X more HCP, mice were morbid with high disease scores and high viral burden (Fig. 6). It is now well appreciated that Delta VoC can cause breakthrough cases in previously infected as well as vaccinated humans (52, 53). Currently, with the highly mutated Omicron VoC, passive immunity and active immunization studies in pre-clinical models will be important to determine the breakthrough capacity of this new VoC. Furthermore, HCP or MAb passive studies can inform the scientific community about enhanced virulence or immune subversion of VoC and we anticipate this passive model can be applied going forward for rapid responses to characterize new variants.

In summary, this study provides insights into differences in SARS-CoV-2 VoC pathogenicity in K18-hACE2 transgenic mice in relation to antibody immunity. Passive immunization of mice with human antibodies can allow for robust characterization of breakthrough capacity (9, 20). This study demonstrates increased disease pathology for mice challenged with Alpha or Beta VoC, and the lack of protection from HCP in mice challenged with Beta or Delta VoC. These data corroborate observations about Beta and Delta VoC in human populations. The human convalescent plasma passive immunity model presented here can be useful in supporting *in vitro* studies and facilitate decision making and planning of research priorities around the overall immune evasion characteristics of SARS-CoV-2 variants.

## MATERIALS AND METHODS

### Ethics and biosafety.

The HCP used in this study was obtained under West Virginia University (WVU) IRB no. 2004976401 (54). HCP was obtained from a single individual with PCR-confirmed SARS-CoV-2 infection in March 2020. Experiments with live SARS-CoV-2 were conducted in Biosafety Level 3 (BSL-3) at Texas Biomedical Research Institute (TBRI IBC BSC20-004) or at WVU (IBC 20-09-03). All BSL-3 animal experiments were conducted under WVU IACUC protocol no. 2009036460.

### Assessment of human IgGs against WA-1 SARS-CoV-2 S RBD and N.

Human IgGs against WA-1 SARS-CoV-2 S RBD and N were quantified using ELISA as described (54). WA-1 S RBD (2 μg/ml) or N (1 μg/ml) proteins were coated on plates and blocked with 3% milk in 0.1% Tween 20 +PBS (PBS-T). Plates were washed three times with PBS-T (200 μl) and virus inactivated samples (25 μl) from human plasma or infected mice were added to 100 μl of sample buffer (1% milk + 0.1% Tween 20 diluted in PBS) and serially diluted (5-fold) down the plates. The final row was left with 100 μl of sample buffer as a negative control. Plates were incubated for 10 min at room temperature shaking at 60 rpm and subsequently washed four times with PBS-T (200 μl). Secondary antibody (100 μl 1:500 anti-human IgG HRP, Invitrogen 31410) was added and plates were incubated for 10 min at room temperature shaking at 60 rpm. After incubation, plates were washed five times with PBS-T (200 μl) and SigmaFAST OPD (Sigma-Aldrich P9187, 100 μl) was added to each well of the plate. OPD development was stopped with 25 μl of 3 M hydrochloric acid and plates were read at an absorbance of 492 nm on a Synergy H1 plate-reader. Binding antibody units (BAU) were calculated based on the NIBSC 1st WHO International Standard (NIBSC code 20/136). Area under the curve analysis was completed in GraphPad Prism v.9.

### Meso Scale Discovery COVID-19 ACE2 Neutralization assay.

SARS-CoV-2 challenged serum was analyzed using the SARS-CoV-2 Plate 11 Multi-Spot 96-well, 10 spot plate following the manufacturer protocol (catalog #: K15458U-2) on the MSD QuickPlex SQ120. The 10 spots contained RBD from different SARS-CoV-2 VoC: (i) B.1427, B.1.429, B.1.526.1; (ii) B.1.351, B.1.351.1; (iii) B.1.525, B.1.526, B.1.618, P.2, R.1; (iv) P.1; (v) B.1.526.2; (vi) B.1.17; (vii) B.1.17+E484K, P.3; (viii) B.1.617, B.1.617.1, B.1.617.3; (ix) AY.3, AY.4, AY.5, AY.6, AY.7, AY.12, AY.14, B.1.617.2, B.1.617.2+Δ144; and (x) A (WT). Three dilutions of serum, 1:10, 1:50 and 1:100 were analyzed for each mouse to perform Area Under the Curve analysis on the electrochemiluminescence using GraphPad Prism v.9.

### Viral growth and *in vitro* analysis of SARS-CoV-2 replication.

SARS-CoV-2 USA-WA-1/2020 (NR-52281) (WA-1), B.1.1.7/Alpha (NR-54000), and B.1.351/Beta (NR-54008) strains were obtained from BEI Resources, and SARS-CoV-2 Delta variant B.1.617.2 hCoV-19/USA/WV-WVU-WV118685/2021 (GISAID Accession ID: EPI_ISL_1742834) was obtained from a patient sample at WVU. These strains were propagated in Vero E6 cells (ATCC-CRL-1586) as described (26, 55). Vero E6 cells for viral titrations (6-well plate, 10^6^ cells/well) were infected with 10-fold serial dilutions of SARS-CoV-2. At 72 h post infection, cells were fixed overnight with 10% formalin (Sigma HT501128-4L), permeabilized and immunostained with 1 μg/ml of a SARS-CoV cross-reactive N protein antibody 1C7C7, kindly provided by Dr. Thomas Moran at the Icahn School of Medicine at Mount Sinai. For viral growth kinetics, Vero E6 cells (6-well plate, 10^6^ cells/well, triplicates) were infected (multiplicity of infection, MOI 0.01) with SARS-CoV-2 WA-1, Alpha or Beta. At the indicated times after viral infection (12, 24, 48 and 72 h), tissue culture samples were collected and titrated by plaque assay as described (26).

### Genome sequencing of SARS-CoV-2 VoC.

SARS-CoV-2 viral RNA from all stocks used for *in vitro* analyses was deep sequenced according to the method described (56). Briefly, we generated libraries using KAPA RNA HyperPrep Kit (Roche KK8541) with a 45 min adapter ligation incubation including 6-cycle of PCR with 100 ng RNA and 7 mM adapter concentration. Samples were sequenced on an Illumina Hiseq X machine. Raw reads were quality filtered using Trimmomatic v0.39 (57) and mapped to a SARS-CoV-2 reference genome (GenBank Accession No. MN985325) with Bowtie2 v2.4.1 (58). Genome coverage was quantified with MosDepth v0.2.6 (59). We genotyped each sample for low frequency VoC with LoFreq* v2.1.3.1 (60) and filtered sites with allele frequencies less than 20%. SARS-CoV-2 viral RNA from stocks used for K18-hACE2 transgenic mouse infection was deep sequenced and reads were aligned to the MN908947.3 reference genome using BWA v.0.7.17 (61) and trimmed for base-calling quality using iVar v.1.3.1 (62) with default parameters. Consensus sequence and individual mutations relative to the reference genome were determined using iVar, with a minimum allele frequency of 30% used as a threshold for calling a mutation. Coverage was computed using samtools mpileup v.1.11 (63). Lineage was confirmed using pangolin v.2.3.5 and pangoLEARN v.2021-03-16 (64). Authentication of the Beta stock was performed using metagenomic sequencing as described (65, 66). Viral RNA was treated with Turbo DNase I (Thermo Fisher). cDNA was generated from random hexamers using SuperScript III reverse transcriptase, second strand was generated using Sequenase 2.0, and cleaned using 0.8× Ampure XP beads purification on a SciClone IQ (Perkin Elmer). Sequencing libraries were generated using two-fifths volumes of Nextera XT on ds-cDNA with 18 cycles of PCR amplification. Libraries were cleaned using 0.8×Ampure XP beads and pooled equimolarly before sequencing on an Illumina NovaSeq (1 × 100bp run). Raw fastq reads were trimmed using cutadapt (-q 20) (67). To interrogate potential resistance alleles, reference-based mapping to NC_045512.2 was carried out using our modified Longitudinal Analysis of Viral Alleles (LAVA; https://github.com/michellejlin/lava) (68) pipeline. LAVA constructs a candidate reference genome from early passage virus using bwa (61), removes PCR duplicates with Picard, calls variants with VarScan (69, 70), and converts these changes into amino acid changes with Annovar (71). The genome sequence for strain Beta is accession number QWE88973. The genome sequence of the Beta contained the expected mutations spike and has a wild type furin cleavage site. A 52aa deletion was observed in orf7a; however, it is not expected that this deletion has any impact on the *in vivo* infection capacity of this strain as orf7a mutants are observed in surveillance. Beta VoC was able to effectively colonize and cause morbidity in experiments presented in this study.

### Challenge of K18-hACE2 transgenic mice with SARS-CoV-2 VoC and treatment with HCP.

SARS-CoV-2 WA-1 and Alpha and Beta VoC were thawed from −80°C and diluted in infection medium (Dulbecco’s Modified Eagle Medium 4/.5g/L glucose + 2% fetal bovine serum + 1% HEPES + 1% penicillin/streptomycin at 100 units/μg/ml) to a concentration of 10^6^ PFU/ml in the WVU BSL-3 facility. Delta VoC was diluted to a 10^4^ PFU/dose from a 2.4 × 10^5^ PFU/ml stock in 1X Dulbecco’s phosphate-buffered saline. Male 8 to 10 weeks old B6.Cg-Tg(K18-hACE2)2Prlmn/J mice (Jackson Laboratory 034860) were anesthetized with a single intraperitoneal dose of ketamine (Patterson Veterinary 07–803-6637, 80 mg/kg) + xylazine (Patterson Veterinary 07–808-1947, 8.3 mg/kg) and 50 μl infectious dose was administered with a pipette intranasally, 25 μl per nare. HCP, 500 μl, or healthy human sera (HHS) with known anti-SARS-CoV-2 IgGs and neutralizing Abs (nAbs) were administered intraperitoneally at this time. For the Delta VoC challenge study, 500 μl HCP was administered for 6 consecutive days (Fig. 6A). Mice were monitored until awake and alert.

### Cumulative disease scoring of SARS-CoV-2 challenged mice.

Mice were scored daily on a scale encompassing appearance (score of 0–2), eye health (score of 0–2), respiration (score of 0–2), activity (score of 0–3) and weight loss (score of 0–5). Appearance included visual identification of a combination of mild to severe piloerection (0–2) or lack of grooming (0–2). Eye health scores were defined by observation of squinting (1), prolonged eye closure not related to sleep (2), or eye discharge (0–2) depending on severity. The maximal combined score for eye health was 2. Respiration (assessed visually) outside the range of 80–240 breaths per minute required mandatory euthanasia and scored as 2. Respiration that was abnormal in regularity was scored as 1. Activity was scored as slow (1), immobile (2), or collapsed and immobile (3). Weight loss was scored as 0–5% (0), 5–10% (1), 10–15% (2), 15–20% (3), >20% (4, 5). All mice with weight loss greater than 20% were humanely euthanized. Rectal temperature was also monitored daily throughout the experiments.

### Euthanasia and necropsy of SARS-CoV-2 challenged mice.

Euthanasia was conducted by administering 200 μl of pentobarbital (Patterson Veterinary 07–805-9296, 390 mg/kg diluted in 0.9% sterile NaCl) and cardiac puncture. Blood was aliquoted into gold serum separator tubes (BD 365967) and centrifuged at 15,000 × *g* for 5 min. Serum was removed and stored in 1.5 ml tubes at −80°C until needed. Lungs were removed from animals and the right lobes of the lung were homogenized in 1 ml of PBS in Miltentyi C tubes (Miltenyi Biotec 130-096-334) using the m_lung_02 program on a Miltenyi gentleMACS tissue dissociator. An aliquot of each lung homogenate (300 μl) was added to 100 μl of TRIReagent (Zymo Research R2050-1-200) and stored at −80°C. Remaining homogenates (300 μl) were spun down at 15,000 × *g* and the supernatants collected. Pellets were frozen at −80°C until use. Brain tissue was removed from animals and split down the mid-line. The right brain was added to 1 ml of PBS in Miltenyi C tubes and homogenized using the m_lung_02 program. An aliquot of each homogenate (500 μl) was added 167 μl aliquots of TRIReagent and stored at −80°C until use. Remaining homogenates were frozen at −80°C until use. To inactivate virus from tissue samples, 1% vol/vol Triton X-100 (Sigma-Aldrich T8787) (27) was added to each sample and incubated for 1 h at room temperature. Inactivated samples were then removed from the BSL-3 High Containment facility.

### Evaluating viral copy number in SARS-CoV-2 challenged tissues.

RNA from homogenized virus-inactivated lung and brain tissues of SARS-CoV-2 infected mice was extracted using the Direct-zol RNA MiniPrep Kit (Zymo Research R2051) following the manufacturer’s instructions. RT-PCR and qPCR were performed by generating a master mix of: 10 μl of TaqMan RT-PCR Mix from the Applied Biosystems TaqMan RNA to CT One Step Kit (Thermo-Fisher Scientific 4392938), 900 nM (1.8 μl) of (ATGCTGCAATCGTGCTACAA) forward nucleocapsid primer (27), 900 nM (1.8 μl) of (GACTGCCGCCTCTGCTC) reverse nucleocapsid primer (27), 250 nM (0.5 μl) of TaqMan probe (56-FAM/TCAAGGAAC/ZEN/AACATTGCCAA/3IABkFQ), 0.5 μl of TaqMan RT enzyme from the Applied Biosystems TaqMan RNA to CT One Step Kit (Thermo-Fisher Scientific 4392938), 100 ng of RNA, and RNase/DNase free water to make a 20 μl total reaction volume. Samples were run in triplicate in Microamp Optical 96-well Fast Reaction Plates (Thermo-Fisher Scientific 4306737) through the following protocol: reverse transcription at 48°C for 15 min, activation of AmpliTaq Gold DNA polymerase at 95°C for 10 min, and 50 cycles of 95°C denaturing for 10 s followed by 60°C annealing for 60 s. Samples were run on an Applied Biosystems StepOnePlus real-time PCR system. Samples with undetectable virus were assigned a value of 1. C_T_ values and copy numbers were calculated and analyzed in Microsoft Excel and GraphPad Prism v.9.0.0.

### Lung histopathology.

Left lobes of lungs were fixed in 10 ml of 10% neutral buffered formalin. Fixed lungs were paraffin embedded into 5-μm sections. Sections were stained with hematoxylin and eosin and sent to iHisto for pathological analysis. Lungs were scored by a pathologist for chronic and acute inflammation in the lung parenchyma, blood vessels, and airways. Pathologist was blinded to the experimental groups but was aware of groups that were challenged with SARS-CoV-2. Each mouse was scored individually using a standard qualitative toxicologic scoring criteria: 0 = none; 1 = minimal; 2 = mild; 3 = moderate; 4 = marked; 5 = severe. Chronic inflammation was marked by lymphocytes, plasma cells, and alveolar macrophages in the parenchyma, blood vessels and airway. Acute inflammation was scored by the presence of neutrophils and edema in the parenchyma, blood vessels and airway.

### Statistical analyses.

All statistical tests were performed on groups with *n* ≥ 5 in GraphPad Prism v.9.0.0. To compare two-groups, student’s *t*-tests were used. To compare three or more groups, one-way ANOVA (parametric data) or Kruskal-Wallis (non-parametric data) were used followed by Tukey’s (parametric data) or Dunn’s (non-parametric data) multiple comparisons tests. To compare grouped data, two-way ANOVA with no correction was performed followed by Tukey’s multiple comparison test. To assess statistical differences between Kaplan-Meyer curves, Mantel-Cox log-rank tests were performed.
